# Elevation of tumour markers TGF-β, M_2_-PK, OV-6 and AFP in hepatocellular carcinoma (HCC)-induced rats and their suppression by microalgae *Chlorella vulgaris*

**DOI:** 10.1186/s12885-017-3883-3

**Published:** 2017-12-21

**Authors:** Khaizurin Tajul Arifin, Suhaniza Sulaiman, Suhana Md Saad, Hanafi Ahmad Damanhuri, Wan Zurinah Wan Ngah, Yasmin Anum Mohd Yusof

**Affiliations:** 1 Department of Biochemistry, Faculty of Medicine, Universiti Kebangsaan Malaysia Medical Centre, Jalan Yaacob Latiff, 56000 Cheras, Kuala Lumpur, Wilayah Persekutuan Malaysia; 2 Department of Diagnostic & Allied Health Sciences, Faculty of Health & Life Sciences, Management & Science University (MSU), University Drive, Seksyen 13, 40100 Shah Alam, Selangor Malaysia

**Keywords:** *Chlorella vulgaris*, Liver cancer, AFP, TGF-β, M_2_-PK, OV-6

## Abstract

**Background:**

*Chlorella vulgaris* (ChV), a unicellular green algae has been reported to have anticancer and antioxidant effects. The aim of this study was to determine the chemopreventive effect of ChV on liver cancer induced rats by determining the level and expression of several liver tumour markers.

**Methods:**

Male Wistar rats (200–250 g) were divided into 4 groups according to the diet given: control group (normal diet), ChV group with three different doses (50, 150 and 300 mg/kg body weight), liver cancer- induced group (choline deficient diet + 0.1% ethionine in drinking water or CDE group), and the treatment group (CDE group treated with three different doses of ChV). Rats were killed at 0, 4, 8 and 12 weeks of experiment and blood and tissue samples were taken from all groups for the determination of tumour markers expression alpha-fetoprotein (AFP), transforming growth factor-β (TGF-β), M_2_-pyruvate kinase (M_2_-PK) and specific antigen for oval cells (OV-6).

**Results:**

Serum level of TGF-β increased significantly (*p* < 0.05) in CDE rats. However, ChV at all doses managed to decrease (*p* < 0.05) its levels to control values. Expressions of liver tumour markers AFP, TGF-β, M_2_-PK and OV-6 were significantly higher (*p* < 0.05) in tissues of CDE rats when compared to control showing an increased number of cancer cells during hepatocarcinogenesis. ChV at all doses reduced their expressions significantly (*p* < 0.05).

**Conclusions:**

*Chlorella vulgaris* has chemopreventive effect by downregulating the expression of tumour markers M_2_-PK, OV-6, AFP and TGF-β, in HCC-induced rats.

## Background

Cancer formation is a complex process involving several stages, namely initiation, promotion and progression [1, 2]. These stages are described as a series of successive mutations that ultimately lead to malignant tumour growth [3]. Increasing evidences showed that accumulation of free radicals in the body causes a variety of biochemical and physiological abnormalities associated with cardiovascular disease, cancer and the aging process [4–6]. Mutation of tumour suppressor genes and activation of protooncogenes would transform normal cells into cancer cells which grow rapidly and metastasize to other parts of the body [7]. To preserve the integrity of an organism, the production of free radicals must be kept in balance by antioxidants. Antioxidants are enzymes [8] or non-enzymes mainly found in our diet that can either scavenge free radicals directly, or regulate its level [9–12].

Fortunately, the presence of most human cancers can be detected by tumour markers, which should have high specificity and few false positive for specific tumours. They must be undetectable in non-neoplastic conditions. Effective screening strategies using molecular markers for most types of cancers have saved or improved quality of life for many patients. Alpha-fetoprotein (AFP) is the most commonly used tumour marker for hepatocellular carcinoma (HCC) screening [13] whereby patients with a high AFP level indicates a bad prognosis than patients with lower AFP levels [14]. Sangiovani et al. (2001) [15] found that cirrhosis patients with elevated AFP level have higher risk of developing HCC. Increased level of AFP in the serum has also been reported to increase in chronic hepatitis B patients [16], fatty liver disease [17] and metabolic syndrome [18]. Although AFP is considered as the gold standard marker for HCC, it is however not useful in the early diagnosis of the disease, particularly AFP-negative HCC, suggesting that new biomarkers are needed [19]. Other liver tumour markers of interest are pyruvate kinase isoenzymes M_2_-PK, transforming growth factor – β (TGF-β), and specific antigen for oval cells, OV-6. M_2_-PK is highly expressed in oval cells, the precursor of liver tumour and it catalyzes the conversion of phosphoenolpyruvate to pyruvate [20]. When a normal cell transforms into a tumour cell, M_2_- PK expression is upregulated due to the uncommonly high anaerobic glycolysis [21], followed by its release into the blood fluid system, which enables it to be measured quantitatively [22]. TGF- β has been recently considered as a possible liver tumour marker. TGF-β1 and TGF-β1 mRNA were shown to be sensitive indicators in the diagnosis of HCC induced by HBV, with the sensitivity and specificity being 89.5 and 94.0%, respectively [23]. Oval cells were first discovered by Farber [24]. They are oval-shaped cells that are not present in the normal liver [25, 26], but their levels are increased during liver regeneration and early stage of HCC. The specific tumour marker for oval cells is OV-6 [27].

*Chlorella vulgaris* (ChV), a unicellular green algae, has long been used as a health supplement especially in Japan and Korea [28, 29]. ChV contains high content of nutrients including vitamins and minerals [30]. It has been shown to strengthen the immune system [31, 32], and exhibit anti- inflammatory effect [32, 33]. In addition, our laboratory has shown that ChV reduced cellular proliferation and induced apoptosis in hepatoma cell line, HepG-2 [34–36], and exhibited antioxidant property in hepatoma-induced rats [35] and STZ-induced diabetic rats [37, 38].

The main objective of this study is to evaluate the antitumour property of ChV in liver cancer- induced rats, by determining the level and expression of novel tumour marker AFP, and comparing with other possible markers M_2_-PK, OV-6 and TGF-β.

## Methods

### *Chlorella vulgaris* culture

*C. vulgaris* algae Beijerinck 072 obtained from UMACC (University of Malaya Algae Culture Collection) Malaysia was grown in Bold Basal Media in laboratory setting with 12 h of dark and light cycle, and harvested by centrifugation (3000 RPM, 10 min, three times at 4 °C). The pelleted algae were diluted in distilled water to arrive at concentrations of 50, 150 and 300 mg/kg body weight.

### Animals, chemicals and treatment

A total of 144 male Wistar rats (200 to 250 g) were obtained from the Animal Unit, National University of Malaysia, and were lodged in polycarbonate cages in a room with controlled temperature, humidity, and light-dark cycle, housed in the animal house of the Institute for Medical Research (IMR), Malaysia. All experiments were conducted following the guidelines of National Institute of Health for the Care and Use of Laboratory Animals. The study was approved by the Animal Ethics Committee of the National University of Malaysia (Approval number: BIOK/2002/YASMIN/30-SEPTEMBER/082). All animals received adequate human care.

The rats were divided into four groups (6 rats per group): control group (normal diet), ChV group with three different doses (50, 150 and 300 mg/kg body weight), liver cancer- induced (choline deficient diet +0.1% ethionine in drinking water to induce cancer) or CDE group [31, 36, 39], and the treatment group or CDE group treated with three different doses of ChV.

The rats in the control group were given both normal diet and drinking water (normal rat chow) via ad libitum. The rats in the ChV group were administered with only ChV at three different doses (50, 150 and 300 mg/kg body weight), per day via oral gavage. The rats in CDE + ChV group were administered with CDE and ChV at 50, 150 and 300 mg/kg. The duration of the experiment was three months and the rats were sacrificed at 0, 4, 8, and 12 weeks. Animals were anesthetized for liver perfusion procedure prior to excision of the liver. Liver tissue was excised and fixed in formalin and embedded in paraffin for immunohistochemistry analysis. Blood was taken via orbital sinus prior to killing the rats at 0, 4, 8 and 12 weeks for determination of TGF-β.

### Hepatic perfusion

Rats were intraperitoneally anesthetized with Zoletil 50 (0.1 ml/100 g body weight), followed by an injection of 0.2 ml of heparin (25,000 U / ml) into the inferior vena cava. The portal vein was canulated with an intravenous catheter needle (16 G IV catheter, 2:25 in.) for the perfusion. Liver was perfused with phosphate-buffered saline (PBS) for 1 min at a flow level of 10 ml/min, followed by perfusion with 4% paraformaldehyde and 0.1% glutaraldehyde (1:1) for three minutes at room temperature. The liver was then perfused again with PBS for two minutes, and rinsed with PBS. A portion of perfused liver was cut and fixed in 10% formalin followed by tissue processing and embedding in paraffin.

### Determination of serum TGF-β

Blood was obtained via the orbital sinus and collected in a tube, to allow it to set for two hours before centrifugation (3000 RPM, 10 min, 4 °C). Serum obtained was stored at −80 °C. The levels of transforming growth factor-β (TGF-β) in the serum was determined by ELISA (BD Pharmingen, USA), according to the protocol by the manufacturer.

### Immunohistochemistry staining for AFP, M_2_-PK, TGF-β and OV-6

Sequential tissue sections (3 μm) were mounted on poly-L-lysine-coated slides. Archival samples were dewaxed by gradual washings in xylene and then dehydrated in various concentrations of alcohols (100, 80, 60 and 40%). Slides then were incubated in 3% hydrogen peroxide in distilled water to quench endogenous peroxidase activity, after which slides were washed under running water. Antigen retrieval was performed by incubating slides in a preheated Colin jar containing Target Retrieval Solution (TRS) pH 9 (Dako, Glostrup, Denmark) for 20 min in a water bath with temperatures ranging from 95 °C to 99 °C. After thermal treatment, the slides were allowed to cool for 20 min at room temperature. Slides were then washed under running water for three minutes and were placed in Tris-buffered saline (TBS), pH 7.6.

Sections were then incubated for 35 min with primary monoclonal antibody: rabbit anti-human AFP (Dako, USA) at 1:200 dilution, or goat anti-human M_2_-PK (Biodesign, USA) at 1:600 dilution, or mouse anti-rat OV-6 (a gift from Dr. Stewart Sell, USA) at 1:400 dilution or mouse anti-human TGF-β at 1:200 dilution. Reaction products were incubated with horseradish peroxidase-conjugated secondary antibody. Diaminobenzidine (DAB) was used as a chromogenic substrate (LSAB = HRP kit, Dako) to visualize the antibody-antigen reaction. All sections were then counterstained with hematoxylin and mounted with permanent mountant DPX. Sections were visualized under light microscopy for assessment of immunoreactivity.

Human liver cancer known to be positive for AFP expression was included as positive control for AFP, cancer-induced liver tissues from previous experiment were used as positive control for both M_2_-PK and OV-6 immunostaining, and lesion-induced gaster tissue was used as positive control for TGF-β.

### Immunoreactivity Assesment

A researcher with no knowledge of clinicopathologic data on the samples evaluated the slides in a blind fashion. Confirmation of the diagnosis was performed by a pathologist evaluating the same slides independently. Most of the slides were classified similarly by both investigators. Results were expressed in percentage of stained cells over total cells counted from ten different fields. A total of 100 stained or non-stained cells were counted from each field at a 40× objective [40].

### Statistical analysis

Statistical analysis was performed with ANOVA using SPSS program ver.11.0. Results were represented as mean ± SEM with *p* < 0.05 considered as significant difference.

## Results

**Fig. 1:**
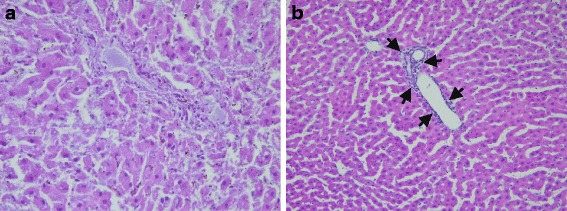
Tissue sections showing rat liver tissues (× 400 magnification). **a**) Normal rat liver tissue. **b**) Formation of oval cells (as shown by arrows) in hepatoma-induced rats

Figure 1b confirms the formation of oval cells as opposed in the control group (Fig. 1a), indicating the cellular changes expected in liver cancer rats. This result shows that rats treated with CDE is a good model for liver cancer rats.

**Fig. 2:**
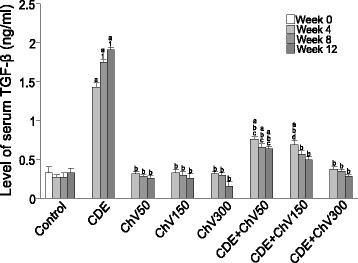
Serum level of TGF-β in CDE rats treated with ChV. ^a^significant difference (*p* < 0.05) compared to control, ^b^significant difference (*p* < 0.05) compared to CDE, ^c^significant difference (*p* < 0.05) compared to ChV50m ^d^significant difference (*p* < 0.05) compared to ChV150, ^1^significant difference (*p* < 0.05) compared to Week 4

Figure 2 clearly showed the hepatoprotective effect of ChV in reducing the elevated levels of TGF-β seen in serum of liver cancer rats (CDE). As seen from the figure, TGF-β level was significantly increased (*p* < 0.05) at all weeks following carcinogen (CDE) diet, compared to control. TGF-β level was significantly higher after eight and 12 weeks (*p* < 0.05), compared to four weeks. Treatment of the rats with ChV at all doses reduced TGF-β level at all weeks. Rats fed with ChV at 300 mg/kg body weight brought the level of TGF-β to almost control level. ChV alone did not raise the levels of TGF-β and seemed to be the same level as in the control, showing no toxicity effect to the liver.

**Fig. 3:**
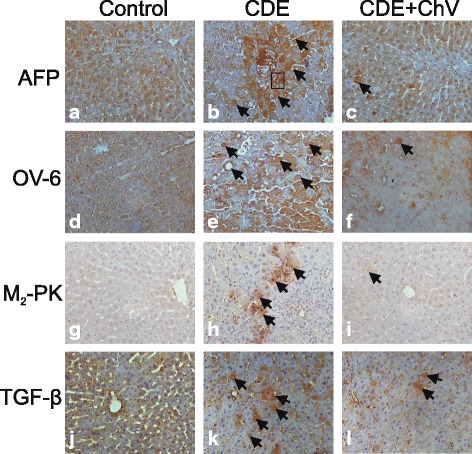
Immunohistochemical expressions of liver tumour markers in rats (× 400 magnification). (**a**) AFP in Control, (**b**) AFP in CDE, (**c**) AFP in CDE + ChV, (**d**) OV-6 in Control, (**e**) OV-6 in CDE, (**f**) OV-6 in CDE + ChV, (**g**) M2-PK in Control, (**h**) M2-PK in CDE, (**i**) M2-PK in CDE + ChV, (**j**) TGF-β in Control, (**k**) TGF-β in CDE and (**l**) TGF-β in CDE + ChV. Arrows indicate brown-stained positive cells. Boxed area in (**b**) contains oval cells

As can be seen from Fig. 3, liver cancer rats resulted in brown-stained cells (shown by arrows in the middle panel) indicating positive expressions of AFP (Fig. 3b), OV-6 (Fig. 3e), M_2_-PK (Fig. 3h) and TGF-β (Fig. 3k), as compared to rats fed with normal diet (Fig. 3a, d, g, and j). Liver cancer rats treated with 300 mg/kg ChV resulted in suppression of the proteins, as evidenced by the smaller amount of brown-stained cells for AFP (Fig. 3c), OV-6 (Fig. 3f), M_2_-PK (Fig. 3i) and TGF-β (Fig. 3l).

**Fig. 4:**
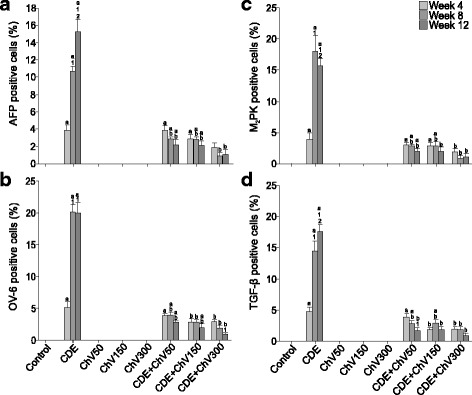
Quantitative expressions of (**a**) AFP, (**b**) OV-6, (**c**) M_2_-PK and (**d**) TGF-β in liver cancer tissues. ^a^significant difference (p < 0.05) compared to control, bsignificant difference (p < 0.05) compared to CDE, ^1^significant difference (p < 0.05) compared to Week 4, ^2^significant difference (p < 0.05) compared to Week 8

Figure 4a shows that the expression of AFP in liver tissues was significantly increased (*p* < 0.05) in the CDE (liver cancer) group for weeks 8 and 12, but treatment with all concentrations of ChV significantly (*p* < 0.05) reduced its expression. Greater reduction of AFP expression was seen at 300 mg/kg body weight (*p* < 0.05).

Similarly, OV-6 (Fig. 4b) was significantly (*p* < 0.05) expressed in CDE group, but showed no significant difference between 8 and 12 weeks of HCC induction. Treatment of the CDE group with 150 and 300 mg/kg body weight ChV significantly reduced (*p* < 0.05) the OV-6 expression for weeks 8 and 12. Treatment with 300 mg/kg body weight ChV for 12 weeks resulted in significant (*p* < 0.05) OV-6 suppression compared to week 4.

M_2_-PK (Fig. 4c) was significantly expressed (*p* < 0.05) in the CDE group, where its expression reached the highest point at 8 weeks, and reduced by more than 2% at 12 weeks. Treatment with 300 mg/kg body weight of ChV resulted in a significant reduction of M_2_-PK expression for all weeks of  supplementation compared to CDE group. ChV at lower doses (50 and 150 mg/kg body weight) significantly (*p* < 0.05) reduced M_2_-PK expression for weeks 8 and 12. 

TGF-β (Fig.4d) was also significantly expressed (*p* < 0.05) in increasing manner in the CDE group with time of exposure to the carcinognen. Its expression peaked at 12 weeks. Treatment with 150 and 300 mg/kg body weight ChV resulted in a significant reduction of TGF-β expression for all weeks of treatment. However a lower dose of ChV (50 mg/kg body weight) significantly reduced TGF-β expression for weeks 8 and 12 only. 

Interestingly the changes of serum level of TGF-β is in concordance with its expressions in tissues (figs. 2 and 4) when treated with ChV. We have also documented the same changes in serum level of AFP in a previous report [41].

## Discussion

Hepatocellularcarcinoma (HCC) affects approximately one million individuals annually worldwide, making it one of the world’s most lethal cancer [42, 43], and the most common form in adults [43]. In 2016, the American Cancer Society estimated there would be over 39,000 of new cases of primary liver cancer and intrahepatic bile duct cancer in the United States alone. Out of this staggering figure, over 27,000 people are estimated to die from these types of cancer [43]. Worldwide, the most common primary liver cancer that occur is HCC, which accounts for 70% to 90% of cases [43].

Thus it is crucial to diagnose HCC early to improve the survival rate of the patients afflicted with the disease. AFP is a the gold standard tumour marker for HCC and its expression is upregulated during hepatocarcinogenesis, hence the use of AFP as a standard biomarker of liver cancer screening [13, 44]. However the specificity and sensitivity of AFP used in liver cancer screening are not satisfactory [45, 46], although it is useful in HCC surveillance in patients with cirrhosis [47]. The outcome of this research indicate the potentials of OV-6, M_2_-PK and TGF-β as liver tumour biomarkers besides AFP, while it attests the beneficial effect of ChV extract as an anti-cancer treatment.

OV-6 and M_2_-PK have been previously shown to over-express in oval cells following HCC induction by choline-deficient + ethionine (CDE) diet [26, 48, 49]. Oval cells play an important role in the development of HCC [50], where their presence is thought to be one of the first cellular changes in hepatic neoplasia, following exposure of the tissue to chemical carcinogens such as ethionine [39, 51]. Oval cells in hepatocytes are transient in nature whereby when triggered by toxic compounds or insults they will proliferate. However normal hepatocytes do not express oval cells as seen in this study and others [52, 53].

Our earlier study has shown the development of liver cancer nodules [36] and increased serum AFP in rats fed with CDE diet [41]. Here we showed that AFP is detected in the hepatocytes of liver cancer rats. High level of AFP has been found in 60–70% of patients with HCC and is associated with poor prognostic and survival rates in untreated patients [54]. AFP also correlates closely with the growth rate (number of dividing cells) and size of the tumour [55] as well as progressive elevation of alpha fetoprotein in biopsied liver samples of patients with liver cirrhosis and hepatocellular carcinoma [56]. Its re-expression in patients with HCC suggests abnormal or altered liver cell regeneration, or dedifferentiation of hepatocytes into tumour cells [57].

Lowes et al. [58] have documented three types of oval cell population; 1) primitive oval cells that are not expressing AFP, OV-6, CK-19 and π-GST, 2) oval-shaped cells resembling hepatocytes and express AFP but not OV-6, CK-19 and π-GST and 3) oval-shaped cells that resemble duct cells expressing OV-6, CK-19 and π-GST but not AFP. However, in this study, the OV-6 was expressed in the cytoplasm of oval-shaped cells resembling hepatocytes and bile duct cells. These results are supported by studies stating that oval cells are multipotent, where they are able to differentiate into hepatocytes [59] and bile ducts [60]. The increased number of oval cells in CDE rats was depicted by the increase in the expression of OV-6, which is consistent with other studies that observed the increasing number of oval cells is directly proportional to the severity of the disease [25, 58, 61]. Expression of OV-6, which is a specific marker for the presence of oval cells, has been reported in the early stages of liver regeneration in human and animals, either due to injury or inhibition of hepatocyte replication [52, 62]. These cells play an important role in the development of HCC [27]. The presence of oval cell population is deemed to be the first cellular changes that occur in neoplastic liver, following intrusion of toxic substances into the liver, in particular carcinogens such as ethionine [58, 61, 63].

In rats supplemented with the carcinogen N-nitrosomospholine (NNM) and CDE, M_2_-PK expression can be observed in the cytoplasm of oval cells found in the liver tissue [64–66]. However, not all oval cells are M_2_-PK-positive. The outcome depends on the fate of the oval cells; either to differentiate into a hepatocyte or a bile duct cell [59, 60]. In this study, the level of M_2_-PK expression was elevated in liver cancer-induced rats as compared to control, plausibly due to the increase in respiratory rate [21], following the transformation of the cell into a cancerous state [20, 64]. However M_2_-PK may not be selective biomarker for liver cancer since its level in the blood has been observed to be raised in other types of cancer such as lung [67], breast [68], cervical [69], esophagus and gastro [21] cancers.

Antioxidants, especially those derived from plant sources are reported to prevent carcinogenesis through the suppression of cell proliferation, stimulation of apoptosis and scavenging free radicals [28]. Our previous study has successfully shown that ChV (300 mg/kg body weight) significantly reduced the percentage of CDE-induced preneoplastic liver nodules (ranging in size from 0.1 to 0.5 cm) from 100% to 17% [36].

ChV has anti-proliferative as well as apoptosis effects against HepG2 liver cancer cells [34, 36, 70]. In addition, Sulaiman et al. [35] showed that ChV treatment resulted in the decline of superoxide dismutase and catalase activity levels, increased level of glutathione peroxidase activity, and the reduction of malondialdehyde in rats supplemented with CDE diet [35]. They suggested that ChV exerted its chemopreventive effect by replacing or compensating for endogenous antioxidant enzyme activity and inhibiting lipid peroxidation. The study also pointed out that free radicals generated by carcinogenesis were scavenged by ChV, which led to the reduction of oxidative stress, thus reducing the formation of cancer cells.

The role of ChV as anticancer agents can be seen clearly in this study. Based on immunohistochemistry results, AFP, OV-6, M_2_-PK and TGF-β were undetectable in the liver tissue of rats supplemented with ChV, thus implying its non-toxic nature. Interestingly, our study showed that treatment of CDE rats with 300 mg/kg body weight ChV as early as four weeks was adequate to suppress AFP expression, while the lowest dose (50 mg/kg body weight) managed to suppress AFP expression after prolonged supplementation for 12 weeks. The efficacy of ChV is also observed with OV-6 expression where prolonged supplementation with ChV for 12 weeks suppressed its expression. ChV was also able to reduce the expression of M_2_-PK, as was also seen with AFP and TGF-β in liver cancer induced rats. The actual mechanism of ChV as an antioxidant and anticancer agent has yet to be elucidated. This study documented the reduction of oval cells formation by ChV supplementation in the liver cancer-induced rats. ChV contains a variety of antioxidants such as ascorbic acid, tocopherols and reduced glutathione [71], and has the potential as an anticancer agent which can restrict and suppress the growth of initiated clonal cell populations into foci or preneoplastic nodules and HCC [72, 73, 74].

## Conclusions

This study documented the reduction of oval cells formation as well as the expression of tumour markers M_2_-PK, OV-6, AFP and TGF-β in HCC induced rats supplemented with *Chlorella vulgaris* (ChV). Based on this study as well as from our previous studies [36, 38, 41], we postulate that the chemopreventive mechanism of ChV, which is rich in antioxidants, is by scavenging ROS found high in tumour cells, as well as inducing antiproliferative and apoptosis effect resulting in the reduction of neoplastic nodules as reflected by the reduction in tumour markers M_2_-PK, OV-6, AFP and TGF-β.

## Abbreviations

π-GST: π-glutathione
ANOVA: Analysis of variance
CK-19: Cytokeratin-19
ELISA: Enzyme-linked immunosorbent assay
Min: Minute
RPM: Revolutions per minute
SEM: Standard error of mean
